# Education of Criminal Children
*"On the Employment of Farm Schools in the Education and Reformation of Pauper and Criminal Children on the Continent," read by the late Joseph Fletcher, Esq., Hon. Sec. to the Statistical Society, before the Society, Feb. 16.


**Published:** 1852-10-01

**Authors:** 


### EDUCATION OF CRIMINAL CHILDREN.*

The subjoined observations are based on information collected for the Belgian Government by M. Edouard Ducpetiaux, showing the unhappy relationship which exists between the workhouse and the gaol, and the constant dependence of at least 50,000 children and young persons under sixteen upon the public guardianship in the one or the other; the recent experience of the continent was interrogated for better systems of management than the lamentable results

* "On the Employment of Farm Schools in the Education and Reformation of Pauper and Criminal Children on the Continent," read by the late Joseph Fletcher, Esq., Hon. Sec. to the Statistical Society, before the Society, Feb. 16.

which we now realize in regard to these young people give us any assurance that we are pursuing.

"The economy of the farm has of late years been variously employed. 1. In free colonies or farm workhouses (*fermes hospices*), which have failed in Holland, but succeeded in Belgium. 2. In colonies for the repression of adult mendicancy and vagabondage, which have universally failed. 3. In agricultural reform schools, refugees, and home colonies for young paupers, mendicants, vagabonds, orphans and foundlings, deserted children, and those who are contaminated with vice, or in moral danger (moral orphans, as they are expressively called), the number of which establishments is large and constantly on the increase in Germany, Switzerland, Holland, France, and Belgium, while they are but now struggling into permanent existence in England. 4. Agricultural penitentiaries, or correctional and reformatory schools, directed exclusively to the training of children and young persons actually found guilty, or acquitted only as having acted without knowledge, but detained for the purpose of being brought up under wholesome discipline to a stated age. The economical position of all such institutions with reference to society at large, was illustrated by that of the *fermes hospices* of Flanders, which are only now coming into general adoption as farm workhouses for paupers of all ages, generally under the management of *sœurs de charité*, and for the most part springing out of the charitable efforts of individuals and the public to rescue these unfortunates of all ages from being sold at auction, by their several communes, to any bidder who, for a stated term, will take and make what use he can of them; each being knocked down, amidst the coarsest ribaldry, to that competitor who required least from the commune for his or her maintenance; and the young being thus often brought up to professional mendicancy, or worse vices, endured many of the miseries and entailed many of the curses of slavery. In the department of Thielt Roulers, in West Flanders, there were, on the 1st of January, 1851, sixteen of these *fermes hospices*, containing 1052 indigent persons, besides 71 "religious" persons in the management of ten of them, and 22 lay persons in the management of ten also; four having both lay and religious managers. The average daily cost of maintenance to the commune for each poor person was 20 cents. or 2d. per day.

"The first step out of the horrible system of pauper slavery above described, once common in its principal features to the whole continent, is, however, due to the inexhaustible charity of the Protestant Cantons of Switzerland; the first among the men of piety and refinement who could no longer endure to regard it with self-sacrifice being Jean Henry Pestalozzi, of Zuric, who gave his whole life and fortune to efforts which produced great changes in the views entertained of education generally. His work, recommenced with zeal by De Fellenberg, at Hofwyl, in 1799, and still continued by his disciple, Vishli, at Kreutzhingen, near Constance, has been imitated throughout Switzerland in farm schools, chiefly deriving their origin, like the former *hospices* of Flanders, from private beneficence and public subscription, seconded by contributions from the communes and the cantonal governments. These are commonly for children of both sexes, from 30 to 50 and upwards in number, economically managed on the plan of an enlarged peasant family, by a married couple, styled respectively 'housefather' and 'housemother,' the former of whom is their leader in industry and their instructor in school. Having been carefully selected and commonly educated for the duties of this station by the Christian originators of each institution, simplicity, piety, order, and happiness appeared to reign in these institutions, and from 1837 to 1840 the Swiss Society of Public Utility commissioned Mr. Kinatli to study the best means of applying this happy discipline also to reformatory purposes. This object he realized in the latter year, in the establishment of the Reformatory School at Bachtaten, the most peculiar features of which are its being for boys only, and its employment of an enlarged proportion of moral agency by the subdivision of the young people into smaller families, each under its own assistant 'housefather.' The mean cost of maintenance and management in thirteen of these Swiss establishments was found to be 185 francs per annum, or 50 cents (5d.) per day.

"Many of the States of Germany have nearly kept pace with Switzerland in these efforts to rescue from destruction the children thrown physically or morally destitute upon society; and, in Wurtemburg especially, the reform schools date from 1828, and in 1841 amounted to twenty, containing 388 male and 675 female children. They now form a complete system under the guardianship of the State, but with a tendency to overburthen each family with numbers, the average in 1844 having increased to 56 children, of whom 33 were boys and 23 girls, supported at an average cost of 60 florins (of 2s.) per annum. But the most remarkable German institution of this kind is the reformatory school of Hamburgh, called the Kautren Haus, at Hom, under the management of M. Wichern, and from the advanced views of which Mr. Kinatli derived the details of his plan for the organisation of Bachtaten. The 'housefathers' here form a Protestant religious fraternity of normal school students for similar labours and for missionary work, but it appears to be almost impossible to obtain the average cost of its 86 children, with their 14 *employés*, because of the great amount of gifts in kind, but on the sums actually brought to account it is averaged by the former only, no less than 300 francs per annum, while at the Prussian establishment at Dusselthal, with 178 children, it is 180 francs.

"In France and in Algeria there appear now to be 41 new colonies for children and young persons, classed as follows:—

| | No. | Average of Land. | No. of Inmates. | Average daily cost of maintenance. |
|---|---|---|---|---|
| Penitentiary colonies founded and directed by private individuals . . | 12 | 2,988 | 1,933 | 1fl. 18c. |
| Penitentiary colonies directed by the State . . . . . . . . . . | 4 | 1,052 | 384 | 0 77 |
| Colonies of orphan, foundling, deserted, and pauper children . . . | 25 | 8,375 | 1,582 | 0 81 |
| Totals . . . . | 41 | 12,415 | 3,899 | 0 844-11 |

"If we include in the average charge per head, the interest of capital and the rent of land, the average cost of these institutions per head per day, may be analysed as follows:—

| Number of Colonies averaged. | Food. | Clothing, Bedding, Sicknesses. | Establishm., Instruction, & Miscel. | Interest of Capital and Rent. | Gross average. |
|---|---|---|---|---|---|
| 12 Penitentiary colonies founded and directed by individuals . | 41c. | 27c. | 30c. | 30c. | 1fl. 28c. |
| 4 Penitentiary colonies directed by the State . . . . . . | 47 | | 30 | 24 | 1 10 |
| 12 Colonies of orphan, foundling, deserted, and pauper children . | 42 | 19 | 21 | 28 | 1 10 |
| General results . . . | 42 | 22 | 26 | 28 | 1 18 |

"Of these 41 establishments, 18 are directed by laymen, 15 by ecclesiastics or religious bodies, and 8 are under a mixed direction, partly lay and partly religious. Three of the establishments are specially devoted to Protestant children, and two of these receive children of both sexes, but all the rest are exclusively for boys. There is, however, a special establishment for young girls under confinement near to Montpelier, under the name of the Solitude of Nazareth, and managed by M. C. Abbé Comal.

"The oldest of these colonies, that of Neuhof, dates from 1825, but 33 were founded at the much later period—from 1837 to 1848—and 7 have been brought into existence or recognised since the revolution of February, 1848. Since this latter period, on the other hand, three colonies have been suppressed."

Postponing to a future occasion an analysis of the experience of France, Belgium, and England, in the application of farm schools to the reformation of criminal youth, Mr. Fletcher then restricted attention to their employment in these countries in the training of pauper and morally endangered children, arriving at these conclusions :—

1. That the farm schools of the continent, applied to education for the *prevention* of crime, hold a social position precisely analogous to that of our own workhouse schools.

2. That for the children in those schools, as in those of the continent, a training in vigorous, rural industry, and close domestic economy, by means of farm schools, conducted on the principles of a Christian family, will yield the greatest attainable moral vigour, with the least amount of indolence and self-deception.

3. That by far the greater number of the present workhouse schools are now producing converse results; and that we have no experience strongly favourable to regimenting and warding the children in large district palaces, however pleasing their mechanism, while we have ample testimony in favour of the farm school system.

4. That the children at a proper farm school, required to work steadily at all its out-door and domestic duties, as well as their own mental cultivation, will certainly not cost more to the public, if so much, as under the present system, or that of the contemplated district asylum, while the saving in their improved conduct for the future would be very great; and,

5. That to have good preventive schools for the training of the pauper children is the great practical step towards obtaining good *reformatory* schools, for the retraining of criminal children, if this is ever to be realized, on principles well understood and economically applied.

